# Excesso de Mortalidade Hospitalar por Doenças Cardiovasculares no Brasil Durante o Primeiro Ano da Pandemia de COVID-19

**DOI:** 10.36660/abc.20210468

**Published:** 2022-05-24

**Authors:** Anderson da Costa Armstrong, Lucas Gomes Santos, Thiago Cavalcanti Leal, João Paulo Silva de Paiva, Leonardo Feitosa da Silva, Gibson Barros de Almeida Santana, Carlos Alberto de Oliveira Rocha, Thiala Alves Feitosa, Sara Larissa de Melo Araújo, Márcio Bezerra-Santos, Carlos Dornels Freire de Souza, Rodrigo Feliciano do Carmo

**Affiliations:** 1 Universidade Federal do Vale do São Francisco Petrolina PE Brasil Universidade Federal do Vale do São Francisco, Petrolina, PE – Brasil; 2 Universidade Federal de Alagoas Campus Arapiraca – Medicina Arapiraca AL Brasil Universidade Federal de Alagoas – Campus Arapiraca – Medicina, Arapiraca, AL – Brasil; 3 Universidade Federal de Sergipe São Cristóvão SE Brasil Universidade Federal de Sergipe, São Cristóvão, SE – Brasil

**Keywords:** COVID-19, Doenças Cardiovasculares, Mortalidade

## Abstract

**Fundamento:**

A pandemia da COVID-19 tem causado um impacto sobre a mortalidade por várias doenças em todo o mundo, especialmente por doenças cardiovasculares (DCVs). O Brasil é um país de dimensões continentais com diferenças significativas na estrutura de saúde entre seus estados.

**Objetivo:**

Analisar a mortalidade hospitalar por DCV no sistema público de saúde durante o primeiro ano da pandemia por COVID-19 (2020) no Brasil.

**Métodos:**

Este é um estudo ecológico analisando o número absoluto de mortes hospitalares e a taxa de mortalidade hospitalar no Brasil, suas macrorregiões, e unidades federativas. Os dados foram obtidos do Sistema de Informações Hospitalares (SIH) do Ministério da Saúde. O P-escore foi usado para analisar o excesso de mortalidade. O escore compara os eventos observados com os eventos esperados para um dado local e período. O escore-P foi corrigido por um modelo de regressão joinpoint, com um intervalo de confiança de 95% e nível de significância de 5%.

**Resultados:**

Houve 93.104 óbitos hospitalares por DCV no Brasil em 2020, o que representa 1495 menos óbitos (escore-P: -1,58) que o esperado. A região centro-oeste apresentou um escore-P positivo, com um aumento de 15,1% no número de mortes. Dez estados apresentaram um maior número de óbitos em 2020. Ainda, observou-se um excesso de 13,3% de mortalidade hospitalar no país como um todo, e um excesso de mortalidade hospitalar em todas as macrorregiões.

**Conclusões:**

Houve uma diminuição no número absoluto de óbitos hospitalares, bem como um aumento na taxa de mortalidade por DCV no Brasil em 2020, após o início da pandemia por COVID-19.

## Introdução

Os primeiros casos da Doença por Coronavírus 2019 (COVID-19) foram registrados em dezembro de 2019 na China, e a doença rapidamente se disseminou em todo o mundo. Em março de 2020, a COVID-19 foi anunciada como pandemia pela Organização Mundial da Saúde.^1,2^ A transmissão ocorre diretamente entre pessoas ou por contato com superfícies contaminadas, favorecendo assim a rápida propagação do vírus. A COVID-19 pode levar à morte, conforme idade, condição imunológica, e doenças crônicas dos pacientes infectados.^3,4^

No Brasil, o primeiro caso foi confirmado em 26 de fevereiro de 2020, e a primeira morte registrada em 17 de março de 2020.^5^ Em 18 abril de 2021, quase um ano e dois meses após o início da pandemia, o país tinha aproximadamente 13,9 milhões de casos confirmados e aproximadamente 373.000 mortes no Brasil, com uma taxa de casos fatais de 2,7%.^6^ Além disso, desde o início da pandemia, o país vem enfrentando uma crise política e econômica, o que tem dificultado ainda mais o controle da doença.^7,8^

A COVID-19 pode ser assintomática, ou manifestar um amplo espectro de sintomas, incluindo febre, dispneia, tosse, mialgia, anosmia e dor torácica.^6^ Ainda, os pacientes podem apresentar sintomas cardiovasculares, causados ou por um comprometimento cardíaco indireto (por inflamação sistêmica, trombogênese, e aumento na demanda metabólica associada a uma baixa reserva cardíaca), ou por ação direta do patógeno no tecido cardíaco.^9^ Assim, o novo coronavírus pode resultar em lesão miocárdica, arritmia, insuficiência cardíaca, miocardite, e choque, principalmente na presença de doença cardiovascular (DCV) pré-existente.^10-12^

Além disso, medidas não farmacológicas visando diminuir a transmissão da COVID-19 na comunidade afetaram a organização dos serviços de saúde, por exemplo, reduzindo o número de consultas presenciais e o horário de funcionamento dos serviços. Tais medidas também incluíram restrições na mobilidade urbana e recomendações para se buscar atendimento médico somente em caso de extrema necessidade.^13-15^ O comportamento da população também mudou, principalmente devido à preocupação quanto à contaminação pelo novo coronavírus.^14,16^

Vários estudos mostraram uma redução significativa nas internações hospitalares por DCVs, paralelamente a um aumento nas taxas de mortalidade e complicações, em comparação às taxas anteriores à pandemia ou de anos anteriores.^17-22^ No Brasil, um estudo relatou diminuição nas internações hospitalares e aumento na mortalidade por DCV durantes os primeiros meses da pandemia.^23^ Contudo, não existem estudos com dados oficiais abrangendo o todo o ano de 2020.

Em um país de dimensões continentais como o Brasil, é fundamental compreender a situação em cada região para ajudar na tomada de decisões políticas. Assim, o objetivo deste estudo foi investigar a mortalidade hospitalar por DCV dentro do sistema público de saúde brasileiro durante o primeiro ano da pandemia da COVID-19 (2020).

## Métodos

Este é um estudo ecológico analisando o número de óbito hospitalar, taxa de mortalidade hospitalar, e causa de mortes de acordo com o capítulo IX da Classificação Internacional de Doenças (CID-10). Foram consideradas unidades de análise: Brasil, suas macrorregiões, e seus estados (ou unidades federativas). Os dados foram obtidos do Sistema de Informações Hospitalar (SIH) do Ministério da Saúde (http://tabnet.datasus.gov.br/cgi/deftohtm.exe?sih/cnv/nruf.def). O SIH registra todas as internações hospitalares financiadas pelo SUS.

A taxa de mortalidade hospitalar foi calculada usando a seguinte equação:


$$\text{Taxa de mortalidade hospitalar =}\frac{\text{Número de mortes hospitalares por DCV}}{\text{Número de internações por DCV}}\;\times \;100$$

O escore P calcula “excesso de mortalidade” como a diferença em porcentagem entre o número de mortes durante um dado período e a média de mortes durante o mesmo período em anos anteriores. O escore P recomendado (usando o número absoluto de mortes hospitalares) e o escore P adaptado (utilizando taxas de mortalidade hospitalar) foram usados para análise da mortalidade hospitalar, de acordo com as equações seguintes:

Escore P para o número absoluto de óbitos hospitalares:


$$\text{Escore P =}\frac{\text{Número de mortes hospitalares por DCV 2020 - Número esperado de mortes hospitalares por DCV}}{\text{Número esperado de mortes hospitalares por DCV}}\;\times \;100$$

Para o escore P adaptado para a taxa de mortalidade hospitalar:


$$\text{Escore P}=\frac{\text{Taxa de mortalidade hospitalar por DCV (2020) - Valor esperado para a mortalidade por DCV}}{\text{Valor esperado para a mortalidade por DCV}}\;\times \;100$$

Nessas equações, o ‘valor esperado’ refere-se à média dos cinco anos anteriores (2015 a 2019).^24^

Uma vez que o cálculo do valor esperado para o ano de 2020 não considera a tendência temporal do fenômeno, ele pode ser superestimado (se o indicador de tendência é descendente) ou subestimado (se a tendência temporal for ascendente). Por isso, também analisamos a tendência temporal usando o modelo de regressão *joinpoint* com o teste de permutação Monte Carlo (4499 permutações). O modelo permite a classificação das tendências em crescente, descendente ou estacionária, e o cálculo da variação percentual média (APC, *average percentage change*). Foram adotados intervalo de confiança de 95% e nível de significância de 5%.

A APC foi usada para corrigir o número de mortes hospitalares esperadas para 2020, e a taxa de mortalidade hospitalar (%). Nesse processo, foi adotada uma série temporal mensal para o período de cinco anos (2015-2019), totalizando 60 meses. Para obter os valores esperados, foram adotadas as seguintes regras:

Para tendência crescente: valor médio de 2015-2019 + APC

Para tendência decrescente: valor médio de 2015-2019 - APC

Para tendência estacionária: foi usado somente o valor médio

Em seguida, o estudo prosseguiu para a análise descritiva (frequência absoluta e relativa) da mortalidade hospitalar e os escores P do país, das macrorregiões e das unidades federativas. Os resultados foram apresentados considerados todo o ano de 2020, e o período de março a dezembro do mesmo ano, considerando que a COVID-19 foi confirmada no Brasil no final de fevereiro, e a doença se espalhou de março em diante.

Foram usados o programa Microsoft Office Excel® (©2008 Microsoft Corporation), SPSS v.21 (©IBM corporation) e regressão Joinpoint 4.5.0.1 (Instituto Nacional do Câncer – EUA).

O estudo utilizou dados de domínio público, que não permite a identificação dos indivíduos. Por esse motivo, o estudo prescindiu de aprovação do comitê de ética em pesquisa.

## Resultados

**Figura 1:**
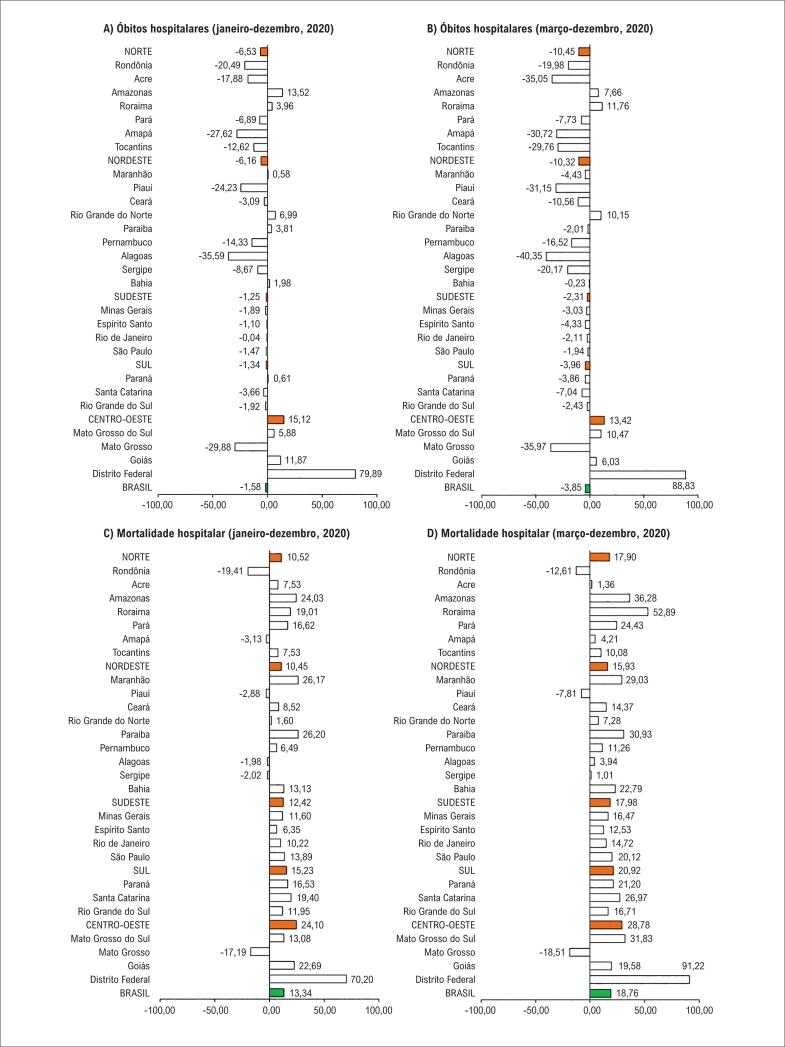
Escore-P para o número absoluto de óbitos hospitalares (A e B) e taxa de mortalidade hospitalar (C e D) por doenças cardiovasculares no Brasil, suas regiões, e unidades federativas durante o primeiro ano da pandemia da COVID-19, Brasil, 2020.

Em 2020, houve 93 104 mortes hospitalares por DCV no Brasil, menos que o número esperado para aquele ano, dado que a média dos cinco anos anteriores (2015 a 2019) foi de 94 599, indicando suma diferença de 1495 mortes hospitalares (escore P: -1,58). Ao se considerar somente os meses de março a dezembro de 2020, esse declínio foi de 3,85% (73 061 mortes hospitalares esperadas e 70 246 observadas). Considerando as macrorregiões, somente a região centro-oeste mostrou um escore P positivo, com um aumento de 15,2% no número de mortes de janeiro a dezembro, e de 13,42% de março a dezembro. Houve 999 mais óbitos em todo o ano de 2020, e 666 mais óbitos considerando somente o período da pandemia (março a dezembro) (Figuras 1 A, B).

Dez unidades federativas mostraram um maior número de mortes em 2020 em relação ao esperado: duas na região norte (Amazonas e Roraima), quatro na região nordeste (Maranhão, Rio Grande do Norte, Paraíba e Bahia), um na região sul (Paraná), e três na região centro-oeste (Mato Grosso do Sul, Goiás, e Distrito Federal). Ao considerar o período de março a dezembro, esse número foi reduzido a seis estados (Amazonas, Roraima, Paraíba, Mato Grosso do Sul, Goiás e Distrito Federal) (Figuras 1 A-B).

Ao analisar a taxa de mortalidade hospitalar de janeiro a dezembro de 2020, observou-se um excesso de 13,34% no Brasil em 2020 (taxa esperada para 2020: 8,28%; taxa observada para 2020: 0.38%). Em relação ao período de março a dezembro, a taxa aumentou de 8,12% para 9,64% (escore P de 18,76). A taxa de excesso de mortalidade também foi analisada em todas as macrorregiões. Os escores P mais elevados foram observados na região centro-oeste (24,10% de janeiro a dezembro e 28,78% de março a dezembro), seguido da região sul (15,23% de janeiro a dezembro e 20,92% de março a dezembro). Ainda, quando todo o ano de 2020 foi analisado, seis unidades federativas apresentaram um p-escore negativo (Rondônia, Amapá, Piauí, Alagoas, Sergipe e Mato Grosso), e ao considerar o período de março a dezembro, três unidades federativas apresentaram um p-escore negativo (Rondônia, Piauí e Mato Grosso) (Figuras 1 C-D).

**Figura 2:**
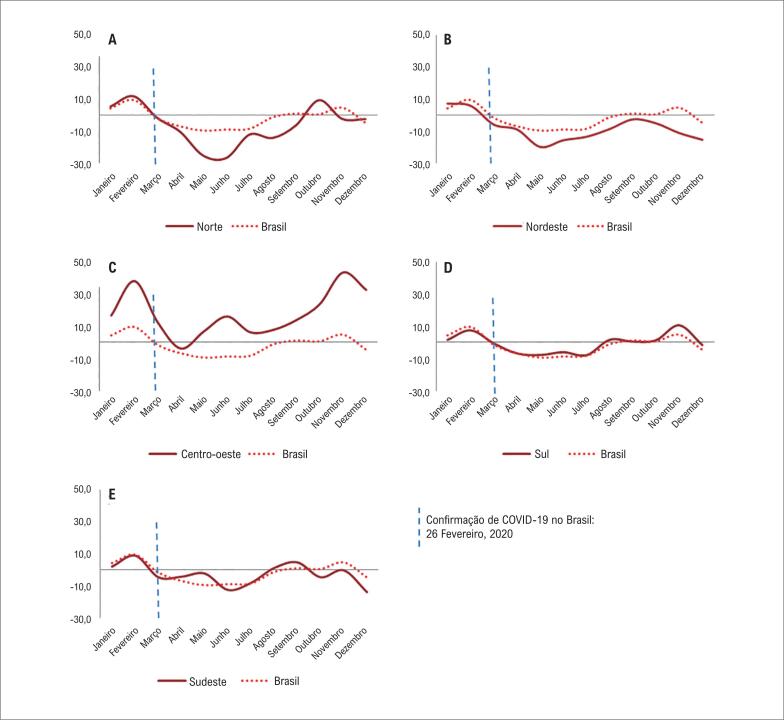
Escore P do número absoluto de óbitos hospitalares por doenças cardiovasculares, por macrorregião, durante o primeiro ano da pandemia da COVID-19, Brasil, 2020.

Durante janeiro e fevereiro, os escores P para mortes hospitalares por DCV no Brasil e suas regiões foram positivos. Em janeiro, por exemplo, o escore P no país foi de 4,4; o escore mais alto foi na região centro-oeste (17,0) e o mais baixo na região sudeste (1,5). Em março, o escore P em todo o país (-1,7) e nas demais regiões (com exceção da região centro-oeste) ficou negativo. O escore P nacional tornou-se positivo de setembro a novembro de 2020. A região nordeste manteve um escore P negativo em todo os meses do ano. Na região sudeste, o escore P tornou-se positivo em agosto (1,5), setembro (0,4), e novembro (10,7) e, no sul, o escore tornou-se positivo em agosto (1,2) e setembro (4,7). Na região centro-oeste, observou-se um padrão peculiar, em que o escore tornou-se negativo somente em abril (-3,7) (Figuras 2 A-E).

**Figura 3:**
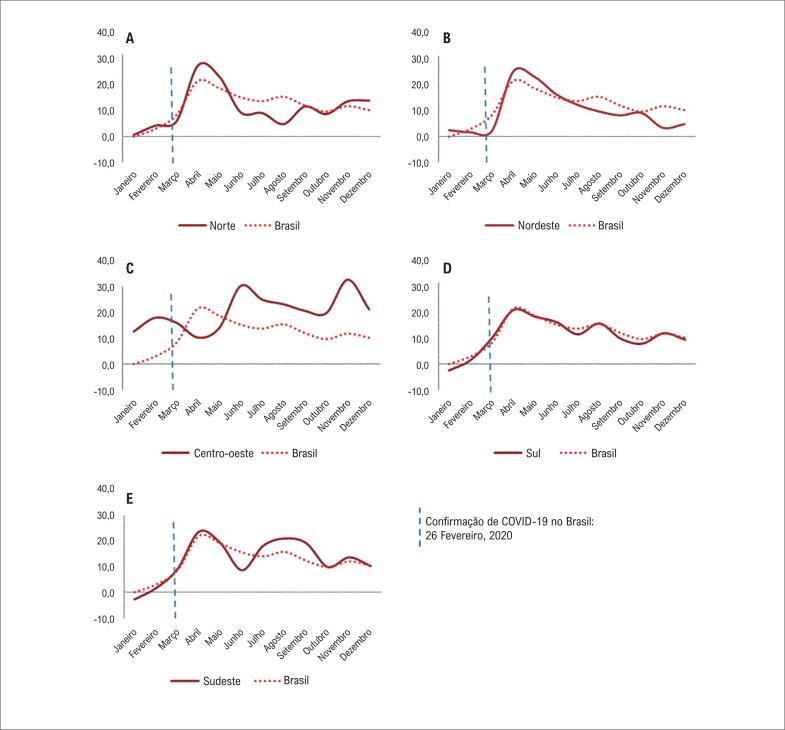
Escore P da taxa de mortalidade hospitalar por doenças cardiovasculares, por macrorregião, durante o primeiro ano da pandemia da COVID-19, Brasil, 2020.

Em janeiro de 2020, foram observados escores P negativos para taxa de mortalidade hospitalar em nível nacional (-0,1), e nas regiões sudeste (-2,9) e sul (-2,5). Por outro lado, a região centro-oeste apresentou um escore P mais elevado (12,7). Para todos os meses subsequentes (fevereiro a dezembro), houve excesso de mortalidade em todas as cinco macrorregiões do Brasil. Destaca-se que, em março, após a pandemia ter sido estabelecida no Brasil, o escore P nacional foi quase três vezes maior que em fevereiro (2,9 vs. 8,9). Ao analisar os dados por região, observou-se que o excesso de mortalidade foi diferente entre as macrorregiões. Enquanto na região sudeste, o escore P aumentou de 1,5 em fevereiro para 10,2 em março (aumento de 6,6 vezes), na região sudeste, o aumento foi de 1,6 para 2,1 (1,3 vezes) na região nordeste, e de 4,4 para 6,1 (1,4 vezes) na região norte. Na região centro-oeste, esse aumento ocorreu mais tarde, somente em maio (Figuras 3 A-E).

## Discussão

Este estudo analisou a mortalidade hospitalar por DCV no sistema público de saúde no Brasil durante o ano de 2020. Observou-se uma diminuição no número absoluto de mortes, além de um aumento na taxa de mortalidade hospitalar em todas as macrorregiões do Brasil e na maioria das unidades federativas no período analisado.

A redução no número absoluto de mortes por DCV no Brasil em 2020 pode ser explicada pelo menor número de pacientes que buscaram os serviços de saúde durante a pandemia,^13^ e a adoção de medidas não farmacológicas para conter a pandemia. Diferentes investigações^17-22^ em todo o mundo relataram um número reduzido de internações hospitalares em 2020 em comparação a períodos anteriores à pandemia, como mostrado em nosso estudo.

Em um estudo multicêntrico no estado de Massachusetts nos EUA, em março de 2020, observou-se uma redução de 43% nas taxas de internações por DCVs agudas, incluindo insuficiência cardíaca, síndrome coronariana aguda, e acidente vascular cerebral.^17^ Outro estudo observou um declínio acentuado no número de admissões hospitalares por outras causas, tais como apendicite aguda, síndrome coronariana aguda, acidente vascular cerebral, fraturas ósseas, câncer, e nascidos vivos, em uma rede de hospitais em Qatar.^25^

Preocupação quanto a contrair COVID-19 nos hospitais,^24^ recomendações de distanciamento social,^26^ e dificuldades de locomoção por meios de transporte público,^27^ podem haver contribuído para a diminuição nas internações e, consequentemente, ao aumento no número absoluto de mortes por DCV registrado em 2020. Estudos brasileiros indicaram que esse cenário epidemiológico contrasta com o aumento nas mortes por parada cardiorrespiratória registradas fora do âmbito hospitalar,^28-30^ como observado na cidade de Belo Horizonte, onde houve um aumento de 33% no primeiro mês da pandemia (março de 2020), em comparação a março do ano anterior.^28^ Ainda, um estudo conduzido na Itália relatou um aumento de 58% em paradas cardiorrespiratórias fora do hospital, e esse aumento esteve fortemente associado com a incidência cumulativa de COVID-19.^31^ Portanto, o que se tem observado é a ocorrência de mortes “ocultas”,^29^ na maioria dos casos, nas casas dos indivíduos que aderiram e respeitaram as recomendações sanitárias.

No Brasil, um país de dimensões continentais, caracterizado por polarização demográfica e epidemiológica,^32^ a pandemia não se espalhou uniformemente pelo território. Consequentemente, a pandemia teve um impacto diferente entre as regiões. Em nosso estudo, com exceção da região centro-oeste, houve diminuição no número de mortes hospitalares por DCV em relação ao esperado em todas as regiões. Ao analisar mês a mês, enquanto foi observado um declínio no P-escore nas regiões norte, nordeste, sul e sudeste no mês de março, na região centro-oeste, essa diminuição ocorreu mais tarde, em abril, e o escore P manteve-se negativo somente nesse mês. Esse resultado está de acordo com a evolução da pandemia nessa região; em março, por exemplo, a região registrava apenas 460 casos da doença, em comparação a 3400 casos registrados na região sudeste.^32^

Dois fatores devem ser considerados em relação à região centro-oeste. É possível que o avanço mais lento da COVID-19 nessa região esteja associado a um menor fluxo migratório de pessoas em comparação a outras regiões como nordeste e sudeste.^33^ Tal fato pode ter adiado o aumento no número de casos de COVID-19 e consequentemente o impacto sobre os serviços de saúde em comparação a outras regiões.^33,34^

Além disso, a região centro-oeste pode ter sido influenciada pelo Distrito Federal, onde o escore P de óbitos foi 81,5, ou seja, bem maior que o esperado. Apesar de não haver uma explicação clara para o alto escore no Distrito Federal, é possível que o fato esteja relacionado a singularidades de seu papel político no país, uma vez que se trata da capital federal do Brasil.^33^ Ainda, existem características locais relacionadas ao sistema de saúde, incluindo a alta disponibilidade de leitos nas unidades de terapia intensiva – 4,5 por 10 000 habitantes (total), 1,6/10 000 habitantes no serviço público, e 11,6/10 000 no setor privado.^35^ A alta disponibilidade de leitos no Distrito Federal também eleva a região centro-oeste à segunda posição em disponibilidade de leitos por macrorregião brasileira (2,5 por 10 000).^35^ Discrepâncias entre unidades federativas em relação à capacidade operacional dos serviços de saúde locais para enfrentar a COVID-19 têm sido um motivo de críticas.^36^ De fato, um estudo conduzido em seis capitais brasileiras mostrou excesso de mortalidade por DCV em cidades menos desenvolvidas durante a pandemia, possivelmente associado ao colapso do sistema de saúde nessas regiões.^37^

Se, por um lado, conforme discutido anteriormente, houve uma redução no número absoluto das mortes hospitalares, por outro lado, observou-se um excesso na mortalidade hospitalar no país como um todo e em todas as macrorregiões. Esse dado está de acordo com o encontrado em estudos anteriores^17-20,22,23^ É pouco provável que esse aumento na mortalidade hospitalar esteja somente relacionado aos efeitos da COVID-19 sobre o sistema cardiovascular. Na Áustria, por exemplo, somente 6,2% dos pacientes admitidos em caráter de urgência por DCV testaram positivo para COVID-19, o que não explicaria o aumento na mortalidade hospitalar em 65% observado nos hospitais daquele país.^18^

Além disso, o aumento na mortalidade hospitalar pode ser um resultado de múltiplos fatores, tais como mudanças no sistema de saúde durante a pandemia. Na Alemanha, um estudo mostrou uma redução nas internações acompanhada por um aumento significativo na mortalidade por infarto agudo do miocárdio durante a pandemia. Os autores também observaram um atraso em se buscar assistência médica desde o início dos sintomas, e pior condição clínica na admissão.^22^ Equipes de saúde têm sido redirecionadas para atender pacientes com COVID-19, e cirurgias eletivas e atendimentos ambulatoriais têm sido interrompidos.^11,23^ Assim, a demora em se buscar atendimento médico,^38-40^ combinada aos efeitos prejudiciais do SARS-CoV-2 no sistema cardiovascular^10,12^ podem haver contribuído para o aumento de descompensação clínica e mortalidade hospitalar durante a pandemia.^11,23^ Um estudo realizado no estado brasileiro de Pernambuco mostrou que a existência de DCV prévia acelerou a mortalidade por COVID-19 em aproximadamente quatro dias.^41^

Em relação às macrorregiões brasileiras, o excesso de mortalidade hospitalar mais elevado (escore P 18,2) foi encontrado na região sul, o que pode ser explicado pelas características demográficas e epidemiológicas da população. Em 2020, 16,4% da população da região tinha idade igual ou superior a 60 anos, e o índice de envelhecimento era de 86% (86 indivíduos com idade igual ou superior a 60 anos para cada grupo de 100 indivíduos com idade menor que 15 anos), o maior do país.^42^ Ainda, a população idosa apresenta a maior prevalência de DCV.^43,44^

### Limitações do estudo

Mesmo considerando a rigidez metodológica adotada, o estudo possui algumas limitações. A primeira diz respeito ao uso de dados secundários do SIH. A qualidade desses dados depende dos registros inseridos no sistema. A qualidade do escore P depende diretamente da acurácia dos dados disponíveis, que pode ser afetada por um atraso entre a ocorrência e o registro do óbito. A falta de dados sobre mortalidade global por doenças cardiovasculares no Brasil, além da avaliação da mortalidade hospitalar restrita aos serviços de saúde pública, são importantes limitações que merecem ser mencionadas.

## Conclusões

O presente estudo mostrou uma diminuição no número absoluto de mortes hospitalares, bem como um aumento na mortalidade hospitalar por DCV no Brasil em 2020, após o início da pandemia por COVID-19, com diferenças entre as macrorregiões e os estados. O efeito da pandemia da COVID-19 tem sido vasto, incluindo um forte impacto sobre os serviços de saúde e doenças existentes. O fortalecimento do sistema de saúde público brasileiro parece ser a medida mais importante para enfrentar a pandemia e suas consequências no país.
