# Supplementary material for: Excesso de Mortalidade Hospitalar por Doenças Cardiovasculares no Brasil Durante o Primeiro Ano da Pandemia de COVID-19
Source: Arq Bras Cardiol. 2022 May 24;119(1):37–45. [Article in Portuguese] doi: 10.36660/abc.20210468 (PMC9352113; doi:10.36660/abc.20210468)
Supplement: Translation

# Translation: In-Hospital Mortality from Cardiovascular Diseases in Brazil during the First Year of The COVID-19 Pandemic

**Authors:** Anderson da Costa Armstrong, Lucas Gomes Santos, Thiago Cavalcanti Leal, João Paulo Silva de Paiva, Leonardo Feitosa da Silva, Gibson Barros de Almeida Santana, Carlos Alberto de Oliveira Rocha, Thiala Alves Feitosa, Sara Larissa de Melo Araújo, Márcio Bezerra-Santos, Carlos Dornels Freire de Souza, Rodrigo Feliciano do Carmo

**Affiliations:** 1 Petrolina PE Brazil Universidade Federal do Vale do São Francisco, Petrolina, PE – Brazil; 2 Arapiraca AL Brazil Universidade Federal de Alagoas - Campus Arapiraca – Medicina, Arapiraca, AL – Brazil; 3 São Cristóvão SE Brazil Universidade Federal de Sergipe, São Cristóvão, SE – Brazil

**Keywords:** COVID-19, Cardiovascular Diseases, Mortality

## Abstract

**Background:**

The COVID-19 pandemic has had an impact on mortality from several diseases worldwide, especially cardiovascular diseases (CVD). Brazil is a continent-sized country with significant differences in the health care structure between its federative units.

**Objective:**

Analyze in-hospital mortality from CVDs in the Brazilian public health system during the first year of the COVID-19 pandemic (2020).

**Methods:**

This is an ecological study analyzing the absolute number of in-hospital deaths and the rate of in-hospital mortality in Brazil, its macro-regions, and federative units. Data were obtained from the Hospital Information System of the Brazilian Ministry of Health. To analyze excess mortality, the P-score was used. It compares the events observed with those expected for a given place and period. The P-score was corrected by the joinpoint regression model, with a 95% confidence interval and 5% significance level.

**Results:**

There were 93,104 in-hospital deaths due to CVD in Brazil in 2020, representing 1,495 fewer deaths (P score: −1.58) than expected. The central-west region had a positive P-score, with a 15.1% increase in the number of deaths. Ten federative units showed a greater number of deaths in 2020. There was also a 13.3% excess in-hospital mortality at the country level, and an excess in-hospital mortality in all macro-regions.

**Conclusions:**

There was a decrease in the absolute number of in-hospital deaths, as well as an increase in in-hospital mortality from CVD in Brazil, in 2020, after the COVID-19 pandemic onset.

## Introduction

The first cases of coronavirus disease 2019 (COVID-19) were registered in December 2019 in China, and the disease quickly spread throughout the world. In March 2020, COVID-19 was declared a pandemic by the World Health Organization.^1,2^ Transmission may be person-to-person or due to contact with contaminated surfaces, thus favoring the rapid spread of the virus. COVID-19 can potentially lead to death, according to age, immune status, and pre-existing chronic diseases of infected patients.^3,4^

In Brazil, the first case was confirmed on 26 February 2020, and the first death was registered on 17 March 2020.^5^ On 18 April 2021, almost one year and two months after the start of the pandemic, there were 13.9 million confirmed cases and approximately 373,000 deaths in Brazil, with a case-fatality rate of 2.7%.^6^ Besides, since the onset of the pandemic, the country has been facing an economic and political crisis, which has made it even more difficult for the country to handle the disease.^7,8^

COVID-19 may be asymptomatic, or manifest a wide spectrum of symptoms, including fever, dyspnea, cough, myalgia, anosmia, and chest pain.^6^ In addition, patients may also present cardiovascular symptoms, either due to indirect cardiac involvement (from systemic inflammation, thrombogenesis, and increased metabolic demand associated with decreased cardiac reserve) or direct action of the pathogen in cardiac tissue.^9^ Thus, the novel coronavirus can result in myocardial injury, arrhythmia, heart failure, myocarditis, and shock, especially in the presence of pre-existing cardiovascular disease (CVD).^10-12^

Furthermore, non-pharmacological measures aimed at reducing COVID-19 transmission at the community level have affected the organization of healthcare services, for instance, by reducing the number of face-to-face consultations and hours of operation of the services. Non-pharmacological measures also included restricted urban mobility and recommendations to seek medical attention only when strictly necessary.^13-15^ Population’s behavior has also changed, primarily due to concerns regarding contamination by the novel coronavirus.^14,16^

Several studies worldwide have demonstrated a significant reduction in hospital admissions due to CVD, in parallel to increased mortality and complication rates, in comparison with rates before the pandemic or previous years.^17-22^ In Brazil, a study reported reduced hospital admissions and increased in-hospital mortality from CVD during the first months of the pandemic.^23^ However, there are no studies with official data from the entire year of 2020.

Moreover, in a continent-sized country like Brazil, it is crucial to understand the situation in each region to help policy decision-making. Therefore, the objective of this study was to investigate in-hospital mortality from CVD within the realm of the Brazilian public health system during the first year of the COVID-19 pandemic (2020).

## Methods

This is an ecological study analyzing the number of in-hospital deaths, rate of in-hospital mortality, and cause of deaths according to chapter IX of the International Classification of Diseases (ICD-10). The following were considered as units of analysis: Brazil, its macro-regions, and its federative units. Data were obtained from the Hospital Information System (SIH, acronym in Portuguese) of the Brazilian Ministry of Health (http://tabnet.datasus.gov.br/cgi/deftohtm.exe?sih/cnv/nruf.def). The SIH registers all hospital admissions financed by the SUS.

The in-hospital mortality rate was calculated using the following equation:


$$\text{In}-\text{hospital mortality rate = }\frac{\text{Number of in}-\text{ hospital deaths due to CVD}}{\text{Number of hospital admissions due to CVD}}\;\times \;100$$

The P-score calculates “excess mortality” as the percentage difference between the number of deaths during a given period and the average number of deaths during the same period in previous years. The recommended P-score (using the absolute number of in-hospital deaths) and the adapted P-score (using in-hospital mortality rates) were used for analysis of in-hospital mortality, as per the following equations:

P-score of the absolute number of in-hospital deaths:


$$\text{P score}=\frac{\text{Number of in}-\text{hospital deaths due to CVD (2020) - Expected number of in - hospital deaths due to CVD.}}{\text{Expected number of in - hospital deaths due to CVD}}\;\times \;100$$

For the adapted P score of in-hospital mortality rate:


$$\text{P Score}=\frac{\text{In}-\text{hospital mortality rate due to CVD (2020) - Expected value of in - hospital mortality due to CVD}}{\text{Expected value of in }-\text{ hospital mortality due to CVD}}\;\times \;100$$

In these equations, the ‘expected value’ refers to the average from the previous five years (2015 to 2019).^24^

Since the calculation of the expected value for the year 2020 does not consider the time trend of the phenomenon, it can be overestimated (if the trend indicator is descending) or underestimated (if the time trend is increasing). For this reason, we also analyzed the period trend by theho joinpoint regression model with Monte Carlo permutation test (4,499 permutations). The model allows the classification of trends as increasing, decreasing or stationary and the calculation of the average percent change (APC). A confidence interval of 95% and a significance level of 5% were adopted.

The APC was used to correct the number of in-hospital deaths expected for 2020, as well as the in-hospital mortality rate (%). In this process, a monthly time series of the five years (2015-2019) was adopted, totaling 60 months. To obtain the expected values, the following rules were adopted:

If increasing trend: mean value of 2015-2019 + APC

If decreasing trend: mean value of 2015-2019 - APC

If stationary trend: only the mean value was used.

Subsequently, the study proceeded to descriptive analysis (absolute and relative frequency) of in-hospital mortality and the P scores for the country, its macro-regions, and federative units. The results were presented considering the whole year of 2020 and the period from March to December of the same year, considering that COVID-19 was confirmed in Brazil at the end of February and the disease spread from March onwards.

The software Microsoft Office Excel® (©2008 Microsoft Corporation), SPSS statistics v.21 (©IBM corporation) e Joinpoint Regression 4.5.0.1 (National Cancer Institute – EUA) were used.

This study used public domain data, which do not allow for identification of individuals. For this reason, approval by the Research Ethics Committee was waived.

## Results

In 2020, there were 93,104 in-hospital deaths due to CVD in Brazil, which is less than that expected for that year, given that the average from the previous 5 years (2015 to 2019) was 94,599, expressing a difference of 1,495 in-hospital deaths (P score: -1.58). When considering only the months from March to December 2020, there was a decrease of 3.85% (73,061 expected in-hospital deaths and 70,246 observed). Regarding the macro-regions, only the Central-West Region showed a positive P score, with a 15.12% increase in the number of deaths from January to December, and 13.42% from March to December. There were 999 more deaths considering the whole year of 2020, 666 more considering only the pandemic period (March-December) (Figures 1 A and B).

**Figure 1:** P-score of the absolute number of in-hospital deaths (A and B) and in-hospital mortality rate (C and D) due to cardiovascular diseases in Brazil, its regions, and federative units during the first year of the COVID-19 pandemic, Brazil, 2020.

Ten federative units showed a higher number of deaths in 2020 in relation to what was expected, as follows: two in the north region (Amazonas and Roraima), four in the northeast (Maranhão, Rio Grande do Norte, Paraíba, and Bahia), one in the south (Paraná), and three in the central-west (Mato Grosso do Sul, Goiás, and the Federal District). When considering the March-December period, this number was reduced to six (Amazonas, Roraima, Paraíba, Mato Grosso do Sul, Goiás and Federal District) (Figures 1 A-B).

When analyzing the in-hospital mortality rate from January to December 2020, an excess of 13.34% was observed in Brazil in 2020 (expected rate for 2020: 8.28%; observed rate for 2020: 9.38%). Regarding the period from March to December, the rate increased from 8.12% to 9.64% (P Score 18.76). Excess in-hospital mortality was also observed in all macro-regions. The highest P-scores were observed in the central-west region (24.10% from January to December and 28.78% from March to December), followed by the south region (15.23% from January to December and 20.92% from March to December). In addition, six federative units showed negative P-score when analyzing the entire year of 2020 (Rondônia, Amapá, Piauí, Alagoas, Sergipe and Mato Grosso) and three when considering the March-December period (Rondônia, Piauí and Mato Grosso) (Figures 1 C-D).

During January and February, the P-scores for in-hospital deaths due to CVD in Brazil and its regions were positive. In January, for example, the nationwide P-score was 4.4; the highest score was in the central-west region (17.0) and the lowest in the southeast (1.5). In March, the nationwide P-score (-1.7) and regional (except for the central-west) P-scores became negative. At the nationwide level, the P-score was observed to become positive from September to November 2020. The northeast region maintained a negative P-score for every month of the year. In the southeast region, the P score became positive in August (1.5), September (0.4), and November (10.7), and, in the South, it became positive in the months of August (1.2) and September (4.7). In the central-west, a peculiar pattern was observed, where the score became negative only in April (-3.7) (Figures 2 A-E).

**Figure 2:** P score for absolute number of in-hospital deaths due to cardiovascular diseases, by macro-region, during the first year of the COVID-19 pandemic. Brazil, 2020.

In January 2020, negative P-scores for in-hospital mortality rate were observed on the nationwide level (-0.1) and in the southeast (-2.9) and south (-2.5) regions. On the other hand, the central-west showed a higher P-score (12.7). For all the following months (February to December), there was excess mortality in all five macro-regions of Brazil. It is noteworthy that, in March, after the pandemic was established in Brazil, the national P-score was almost three times higher (from 2.9 to 8.9) than in February. When analyzing the data by region, it was observed that excess mortality differed between macro-regions. While the P-score increased from 1.5 in February to 10.2 in March (6.6 times greater) in the southeast, it increased from 1.6 to 2.1 (1.3 times greater) in the northeast and from 4.4 to 6.1 (1.4 times greater) in the north. In the central-west, this increase occurred later, only in May (Figures 3 A-E).

**Figure 3:** P score for in-hospital mortality rate due to cardiovascular diseases, by macro-region, during the first year of the COVID-19 pandemic. Brazil, 2020.

## Discussion

This study analyzed in-hospital mortality from CVD in public health in Brazil during the year of 2020. A decrease was observed in the absolute number of in-hospital deaths, in addition to an increase in the in-hospital mortality rate in all Brazilian macro-regions and in most federative units during the period analyzed.

The decrease in the absolute number of deaths due to CVD in Brazil in 2020 may be explained by the lower number of patients seeking health services during the pandemic, and adoption of non-pharmacological measures to contain the pandemic.^13^ Different investigations^17-22^ throughout the world have reported reduced numbers of hospital admissions in 2020, when compared to periods before the pandemic, as was shown in our study.

In a multicenter study in the state of Massachusetts, USA, in March 2020, a 43% reduction was observed in hospitalization rates due to acute CVDs, including heart failure, acute coronary syndrome, and stroke.^17^Another study observed a sharp decline in the number of hospital admissions due to other causes, such as acute appendicitis, acute coronary syndrome, stroke, bone fractures, cancer, and live births, in a network of hospitals in Qatar.^25^

Concerns about contracting COVID-19 in hospital centers,^24^ social-distancing recommendations,^26^ and difficulties in locomotion by means of public transportation,^27^ may have contributed to the decrease in hospital admissions and, consequently, to the decrease in the absolute number of deaths due to CVD registered in 2020. Brazilian studies have indicated that this epidemiological scenario is in contrast with the increase in out-of-hospital deaths due to cardiorespiratory arrest,^28-30^ as observed in the city of Belo Horizonte, where there was a 33% increase in the first month of the pandemic (March 2020), in comparison to March of the previous year.^28^ Furthermore, a study conducted in Italy reported a 58% increase in out-of-hospital cardiac arrest, which was strongly associated with the cumulative incidence of COVID-19.^31^ Therefore, what has been observed is the occurrence of hidden mortality,^29^ mostly at the homes of individuals who adhered to and respected sanitary recommendations.

In Brazil, a continent-sized country, characterized by demographic and epidemiological polarization,^32^ the pandemic has not spread homogenously throughout the territory. Consequently, the pandemic has had a different impact across regions. In our study, except for the central-west, there was a decrease in the number of in-hospital deaths due to CVD in relation to what was expected in all the regions. When analyzing by month, while a decline in P-score was seen in the north, northeast, south, and southeast regions in March, in the central-west, this decrease was observed later, in April, and it remained negative only during this month. This result is in line with the course of the pandemic in this region; in March, for instance, the region registered only 460 cases of the disease, in contrast with 3,400 cases registered in the southeast.^32^

Two factors should be considered with respect to the central-west region. It is likely that the slower advance of COVID-19 in this region is associated with the lower migratory flow of people, as compared with other regions like the northeast and the southeast.^33^ This fact may have led to a delayed increase in the number of cases of COVID-19, and consequently a delayed impact on health services in comparison to other regions.^33,34^

In addition to this, the central-west may have been influenced by the Federal District, where P-score of deaths was 81.5, much higher than the expected. Even though it is not possible to give a clear explanation about this high score in the Federal District, it may be related to the singularities regarding its political role in the country, given that it is the national capital of Brazil.^33^ Also, there are local characteristics related to the health system, including the high availability of intensive care unit beds – 4.5 per 10,000 inhabitants (overall), 1.6/10,000 in the public sector, and 11.6/10,000 in the private one.^35^ This high availability of beds in the Federal District also moves the central-west region to second place in availability of beds by Brazilian macro-region (2.5 per 10,000).^35^ Discrepancies between federative units regarding the operational capacity of their local health services to handle COVID-19 have been a point of criticism.^36^ In line with this, a study conducted in six Brazilian capitals, showed a higher excess mortality from CVD in less developed cities during the pandemic, possibly associated with the collapse of the health system in these regions.^37^

If, on one hand, as previously discussed, there was a reduction in the absolute number of in-hospital deaths, on the other hand, an excess was observed in the in-hospital mortality rate at the country level and in all macro-regions, which is in agreement with what has been observed in previous studies^17-20,22,23^ It is unlikely that this increase in in-hospital mortality is merely related to the effects of COVID-19 on the cardiovascular system. In Austria, for instance, only 6.2% of patients urgently admitted due to cardiovascular conditions tested positive for COVID-19, which would not explain the 65% increase in in-hospital mortality observed in hospitals in that country.^18^

Also, the increase in in-hospital mortality may be the result of multiple factors, such as changes in the health system during the pandemic. In Germany, a study showed a reduction in hospital admissions accompanied by a significant increase in mortality due to acute myocardial infarction during the pandemic. They also observed a greater delay in seeking medical assistance from the onset of symptoms and worse clinical condition upon admission.^22^ Health teams have been redirected to provide care for patients with COVID-19, and elective surgeries and outpatient care have been interrupted.^11,23^ Therefore, the delay in seeking medical assistance^38-40^ and the harmful effects of SARS-CoV-2 on the cardiovascular system^10,12^ may have contributed to increased clinical decompensation and in-hospital mortality during the pandemic.^11,23^ A study conducted in the Brazilian state of Pernambuco showed that prior existence of CVD accelerated mortality from COVID-19 by approximately four days.^41^

Regarding Brazilian macro-regions, the highest excess in-hospital mortality (P-score 18.2) was found in the south, which may be explained by demographic and epidemiological characteristics of the population. In 2020, 16.4% of the region’s population was 60 years or older, and the aging index was 86% (86 individuals aged 60 years or older for each group of 100 individuals under 15 years of age), which is the highest in the country.^42^ Furthermore, the elderly population has the highest prevalence of CVD.^43,44^

### Study limitations

Even considering the methodological robustness of this study, it has some limitations. The first concerns the use of secondary data from the SIH. The quality of these data depends on records inserted into the system. The quality of the P-score depends directly on the accuracy of available data, which means that a delay between occurrence and registration of death can affect its accuracy. The lack of data on overall mortality from cardiovascular diseases in Brazil, in addition to the assessment of in-hospital mortality restricted to the public health network are important limitations that deserve to be mentioned.

## Conclusions

This study has demonstrated a decrease in the absolute number of in-hospital deaths, as well as an increase in in-hospital mortality due to CVD in Brazil in 2020, after the onset of the COVID-19 pandemic, with differences between macro-regions and federative units. The impact of the COVID-19 pandemic has been vast, including a profound effect on health services and existing diseases. Strengthening the national public health system seems to be the most important measure in handling the pandemic and its consequences in Brazil.
